# Can Overnutrition Lead to Wasting?—The Paradox of Diabetes Mellitus in End-Stage Renal Disease Treated with Maintenance Hemodialysis

**DOI:** 10.3390/nu14020247

**Published:** 2022-01-07

**Authors:** Krzysztof Hoppe, Krzysztof Schwermer, Mikołaj Dopierała, Małgorzata Kałużna, Anna Hoppe, Jadzia Tin-Tsen Chou, Andrzej Oko, Krzysztof Pawlaczyk

**Affiliations:** 1 Department of Nephrology, Transplantology and Internal Diseases, Poznan University of Medical Sciences, 60-355 Poznań, Poland; kschwermer@ump.edu.pl (K.S.); dopierala.mikolaj@spsk2.pl (M.D.); jadzia.chou@gmail.com (J.T.-T.C.); aoko@ump.edu.pl (A.O.); 2 Department of Endocrinology, Metabolism and Internal Diseases, Poznan University of Medical Sciences, 60-355 Poznań, Poland; mkaluzna@ump.edu.pl; 3 Department of Haematology and Bone Marrow Transplantation, Poznan University of Medical Sciences, 60-569 Poznań, Poland; anna.kawka@skpp.edu.pl

**Keywords:** end-stage renal disease (ESRD), hemodialysis (HD), protein energy wasting (PEW), diabetes mellitus (DM), bioelectrical impedance spectroscopy (BIS), Subjective Global Assessment (SGA), mortality, dietary intake, body composition, obese sarcopenia

## Abstract

Background: The population of end-stage renal disease (ESRD) patients with diabetes mellitus (DM) may be at increased risk of protein energy wasting (PEW). The aim of the study was to investigate the impact of DM on selected indicators of PEW in the ESRD population that was undergoing maintenance hemodialysis (MHD). Methods: A total of 515 MHD patients were divided into two subgroups with and without DM. The evaluation of diet composition, Charlson Comorbidity Index (CCI), SGA, and laboratory and BIS analyses were performed. All-cause and cardiovascular mortality was recorded. Results: DM patients had lower albumin (3.93 (3.61–4.20) vs. 4.10 (3.80–4.30) g/dL, *p* < 0.01), total cholesterol (158 (133–196) vs. 180 (148–206) mg/dL, *p* < 0.01), and creatinine (6.34 (5.08–7.33) vs. 7.12 (5.70–8.51) mg/dL, *p* < 0.05). SGA score (12.0 (10.0–15.0) vs. 11.0 (9.0–13.0) points, *p* < 0.001), BMI (27.9 (24.4–31.8) vs. 25.6 (22.9–28.8) kg/m^2^, *p* < 0.001), fat tissue index (15.0 (11.4–19.6) vs. 12.8 (9.6–16.0) %, *p* < 0.001), and overhydration (2.1 (1.2–4.1) vs. 1.8 (0.7, 2.7) L, *p* < 0.001) were higher in the DM group. Increased morbidity, reflected in the CCI and mortality—both all-cause and cardiovascular—were observed in DM patients. Conclusions: Hemodialysis recipients with DM experience overnutrition with a paradoxically higher predisposition to PEW, expressed by a higher SGA score and lower serum markers of nutrition. This population is also more comorbid and is at higher risk of death, including from cardiovascular causes.

## 1. Introduction

Diabetes mellitus (DM) is the most common cause of chronic kidney disease (CKD) and its final irreversible stage, end-stage renal disease (ESRD); up to 90% of ESRD patients with a diabetic background receive renal replacement therapy (RRT) via in-center maintenance hemodialysis (MHD) [1]. Most of the diabetic patients treated with MHD suffer from type 2 DM, which is associated with hyperalimentation, obesity, and metabolic syndrome [2]. Patients with ESRD, especially the population treated with MHD, have increased cardiovascular (CV) complications as the main causes of hospitalizations and mortality [1,3]. Although CV risk in the general population is increased in the course of obesity and DM, the CV complications experienced by patients on MHD are frequent and may be life-threatening due to a kidney disease-derived wasting syndrome [2,4]. Kidney disease wasting was historically described as general malnutrition; however, its complex uremic etiology required that it be distinguished from basic abnormalities resulting from an inadequate diet [5,6]. Therefore, the International Society of Renal Nutrition and Metabolism proposed the term “protein energy wasting” (PEW) to solve the significant nomenclatural inconsistencies [4]. PEW results from numerous mechanisms such as uremic toxicity, metabolic and hormonal dysregulations, the RRT modality and its adequacy, comorbidities, supportive treatment as well as psychosocial aspects such as the patient’s nutritional practices, physical activity, and severity of depression [7,8].

Various methods have been proposed for the thorough assessment of PEW in CKD, and their employment has led to the establishment of the clinical diagnostic criteria [7] based on serum markers (e.g., albumin < 3.8 g/dL or pre-albumin < 30 mg/dL, decrease in serum creatinine and total cholesterol over time on dialysis), body composition derangement (e.g., BMI < 23 kg/m^2^, reduction of body weight and fat percentage, muscle mass loss percentage, reduced mid-arm muscle circumference), and a deficient diet based on the recommended protein and caloric intake [7,9].

Bioelectrical impedance spectroscopy (BIS) is an additional technique in the detection of PEW, which has been proven to be valuable. Bioimpedance analysis (BIA) allows for the estimation of lean and fat tissue body mass as well as the determination of body fluid content and its localization [10,11,12]. The assessment of the aforementioned non-laboratory indicators of PEW is achieved through scoring systems [7,9]. One such scoring system is the Subjective Global Assessment (SGA) with a dialysis-modified questionnaire, which notes the patient’s weight change over the last six months, dietary intake, gastrointestinal symptoms, functional capacity due to nutritional impairment and comorbidities, combined with anthropometric measurements to assess fat tissue loss and muscle wasting. Each category is given a score of 1–5 points so that the total value oscillates between 7 (suggesting a proper nutritional state) and 35 (reflecting severe malnutrition) [13].

Both PEW and DM are important risk factors of CV and the overall mortality of MHD patients, thus making it necessary to analyze their potential relationship in order to improve future therapeutic approaches [1,2,6,14,15]. Nevertheless, due to the complex background and the absence of a single universal reliable diagnostic method of PEW in the assessment of CKD, its hitherto diagnostics remain deficient. Therefore, new studies introducing and comparing multiple methods of PEW determination are of great clinical value. Moreover, previous studies on MHD patients’ nutritional state with the implementation of the different diagnostic criteria mentioned above have indicated DM as a risk factor of PEW in ESRD [16,17,18,19]. These observations require in-depth analyses.

The aim of this study was to investigate the impact of DM on selected indicators of PEW in the population of ESRD patients treated with MHD.

## 2. Materials and Methods

### 2.1. Study Design

The study protocol was approved by the Ethics Committee of Poznan University of Medical Sciences (461/15).

**Figure 1:**
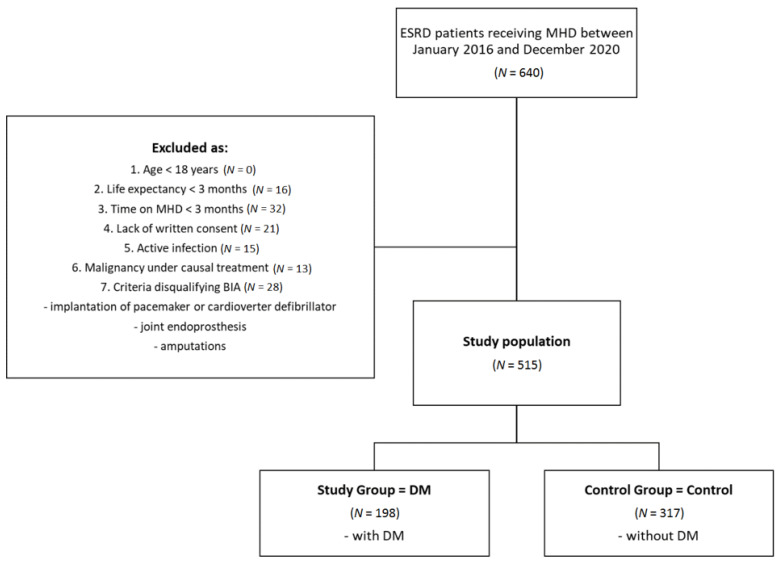
Patient selection flowchart. DM = diabetes mellitus; ESRD = end-stage renal disease; MHD = maintenance hemodialysis.

This prospective, multicenter, observational study was performed in a cohort of 515 ESRD patients (310 males; 205 females; median age 67.3 (58.0–79.4)) who were treated with MHD. The exclusion criteria were: age less than 18 years, life expectancy < 3 months, time on MHD < 3 months, active infection or malignancy under causal treatment, lack of written consent, or at least one of the criteria disqualifying BIA (implantation of pacemaker, cardioverter defibrillator, or joint endoprosthesis and amputations). Patients were divided into 2 age- and sex-matched subgroups: DM (group with DM) and Control (control group without DM). A flowchart depicting patient selection is presented in Figure 1.

During the initiation process, all subjects received a detailed study description and an informed consent form. The study protocol included the full interview, noting residual daily diuresis, use of alcohol and cigarettes, additive salt, comorbidities (hypertension (HT), chronic heart failure (CHF), coronary artery disease (CAD), myocardial infarction (MI), cerebrovascular incident (CVI), atrial fibrillation (AF)), with the application of the Charlson Comorbidity Index (CCI) and SGA [20]. The physical examination included blood pressure (BP) determination and the anthropometric measurements contained in the SGA. A certified dietician performed the quantitative evaluation of diet composition in terms of the intake of energy and different nutritional components: proteins, lipids, cholesterol, carbohydrates, fiber, sodium, and potassium. Total metabolic rate, as an indicator of adequate nutritional requirements for each patient, was calculated based on body weight, height, age, sex, and activity level, i.e., resting metabolic rate using the Mifflin-St. Jeor equation multiplied by the physical activity ratio [21]. Patients prepared a 3-day food diary, including one hemodialysis day and 2 non-dialysis days [22]. Food intake expressed in grams was converted to nutrient intake using a dietary calculator based on the USDA National Nutrient Database for Standard Reference [23]. The study protocol also included a BIA assessment of body composition and a blood sample. Mortality, with specification into CV and non-CV causes, was recorded during the follow-up.

### 2.2. Measurements

All measurements were performed shortly before a mid-week hemodialysis session. The BIA was performed with a Body Composition Monitor (BCM; Fresenius Medical Care Deutschland GmbH, Bad Homburg, Germany) to determine the following parameters of body composition and hydration status: lean tissue mass (LTM), lean tissue index (LTI), fat tissue mass (FTM), fat tissue index (FTI), overhydration (OH), and relative overhydration (OH%) [10]. BIA was assessed in a supine position using disposable electrodes, which were attached to the hand and foot, contralateral to the arteriovenous fistula.

Blood samples were obtained to evaluate serum albumin, creatinine, and total cholesterol as laboratory markers of nutritional status. Additional measurements were made of complete blood count, high sensitivity cardiac troponin T (hsTnT), sodium, potassium, C-reactive protein (CRP), and markers of bone metabolism (calcium, phosphate, intact parathormone) and iron metabolism (iron, total iron binding capacity (TIBC), transferrin saturation (TSAT), ferritin).

### 2.3. Statistical Analysis

Statistical calculations were performed using STATISTICA v. 13.3 (StatSoft, Inc., Tulsa, OK, USA). Descriptive statistics for patients and disease characterization were used. The Shapiro–Wilk test was used to determine variable normality distribution. Results were expressed as medians (first quartile (Q1)—third quartile (Q3)) due to the lack of normality. Differences in nominal variables were assessed with the χ2 test, while the significance of correlations was evaluated with the Spearman test. The comparison of two independent subgroups was performed using the Mann–Whitney test. Survival analysis was completed with the Kaplan–Meier survival curve and the age- and sex-adjusted Cox proportional hazard model. Cox regression analyses with the inclusion of clinical and biochemical confounders as multivariable covariates are presented as hazard ratios with 95% confidence intervals. A *p*-value below 0.05 was regarded as statistically significant.

## 3. Results

### 3.1. Characteristics of the Studied Subgroups

Demographics and data obtained from the interview and physical examination of the studied subgroups are presented in Table 1.

**Figure 2:**
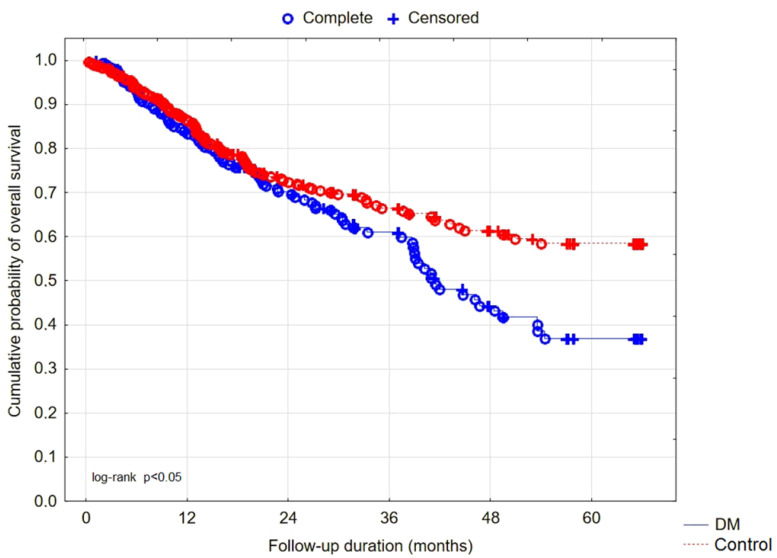
Kaplan–Meier curves for overall survival. The number of patients at risk, number of patients censored, number of events, and cumulative survival at follow-up every 12 months for the DM and Control groups, respectively.

Of the 515 patients included in the study, 198 were assigned to the DM group and 317 to the Control group. In the DM group, nine patients had type 1 DM, and 189 had type 2 DM. There were no significant differences in median age, duration of MHD, gender distribution, alcohol, cigarette, and additive salt use. A higher percentage of the DM group suffered from CV diseases compared to Control, namely CHF (60.6 vs. 45.7%, *p* = 0.017), CAD (63.6 vs. 45.1%, *p* < 0.001), MI (32.8 vs. 18.3%, *p* < 0.001), CI (18.7 vs. 7.9%, *p* < 0.001), and displayed a greater tendency towards HT (92.9 vs. 84.5%, *p* = 0.053). The incidence of AF, median residual daily diuresis, and pre-HD BP were comparable. Increased morbidity reflected by the CCI (5.0 (4.0–6.0) vs. 3.0 (2.0–4.0) points, *p* < 0.001) and mortality, both all-cause (44.9 vs. 31.2%, *p* < 0.01) and CV (29.8 vs. 18.9%, *p* < 0.01), were observed in DM patients. Lower overall survival for the DM group was also illustrated with Kaplan–Meier curves (Figure 2). The median SGA score was higher in the DM group than Control (12.0 (10.0–15.0) vs. 11.0 (9.0–13.0) points, *p* < 0.001). Anuria, DM, age, and SGA appeared as the risk factors of death, whereas albumin, hemoglobin, and BMI were inversely associated with mortality. The Cox proportional hazard model is shown in Table 2.

### 3.2. Laboratory Analysis

Laboratory results are presented in Table 2. Median concentrations of serum markers of nutrition—albumin (3.93 (3.61–4.20) vs. 4.10 (3.80–4.30) g/dL, *p* < 0.01), total cholesterol (158 (133–196) vs. 180 (148–206) mg/dL, *p* < 0.01), creatinine (6.34 (5.08–7.33) vs. 7.12 (5.70–8.51) mg/dL, *p* < 0.05) were lower, while median hsTnT (54 (37–101) vs. 48 (29–81) ng/mL, *p* < 0.05) was higher in the DM group. A higher percentage of patients with hypoalbuminemia—defined as serum albumin below 3.8 g/dL—was detected in the DM group (26.7 vs. 23.7%, *p* < 0.05). No significant differences in other laboratory parameters were detected (Table 3).

### 3.3. Body Composition Based on Bioimpedance Analysis

Median BMI (27.9 (24.4–31.8) vs 25.6 (22.9–28.8) kg/m^2^, *p* < 0.001), FTM (31.6 (26.3–38.5) vs. 25.9 (19.7–33.3) kg, *p* < 0.001), FTI (15.0 (11.4–19.6) vs. 12.8 (9.6–16.0) kg/m^2^, *p* < 0.001), OH (2.1 (1.2–4.1) vs. 1.8 (0.7–2.7) L, *p* < 0.001), and OH% (3.0 (1.5–5.9) vs. 2.5 (1.0–4.2) %, *p* < 0.01) were higher in the DM group. The median values of LTM and LTI were comparable in both subgroups. A higher percentage of patients with BMI < 23 kg/m^2^ was observed in the Control group. A comparison of body composition parameters is presented in Table 4.

### 3.4. Nutritional Components Evaluation

The median intake of calories (2668 (2456–2760) vs. 2279 (2122–2611) kcal, *p* < 0.001), proteins (92 (81–102) vs. 79 (69–89) g, *p* < 0.01), lipids (82 (79–87) vs. 75 (68–82) g, *p* < 0.01), cholesterol (352 (321–398) vs. 309 (221–365) mg, *p* < 0.05), and carbohydrates (376 (346–403) vs. 321 (308–375) g, *p* < 0.001) was higher among the subjects from the DM group. The requirements for the following nutritional components—energy (107.8 (97.1–115.1) vs. 96.7 (89.7–105.3) %, *p* < 0.05), proteins (112.2 (100.1–120.9) vs. 96.4 (85.1–110.2) %, *p* < 0.01), and carbohydrates (109.9 (97.2–116.5) vs. 99.5 (89.8–106.8) %, *p* < 0.05)—were met in higher percentages in the DM group. No differences in the intake of other nutritional components were detected. The details are presented in Table 5.

### 3.5. Correlations between cTnT and Markers of Nutritional State

The correlations were performed to identify the most useful nutritional state indicators as markers of CV risk. In the DM group, cTnT correlated positively with SGA and albumin, while in the Control group, cTnT correlated positively with the same parameters and negatively with BMI, FTI, and total cholesterol. The details are presented in Table 6.

## 4. Discussion

Our study is one of the few focused on the association between DM and the markers of nutritional state among patients undergoing MHD therapy. ESRD is a deteriorating catabolic condition predisposing patients to PEW, which is in stark contrast to type 2 DM, a systemic metabolic disease associated with overnutrition and obesity [2,4]. This study intended to investigate the potential paradox of DM as a risk factor of PEW development in ESRD patients receiving MHD. To achieve this, we performed complex analyses of different indicators of the nutritional state and body composition of age- and sex-matched populations with and without DM. Our analysis was based on four diagnostic fields. The first was the dietary intake of energy and different nutritional components according to their optimal requirements. The second was the dialysis-modified questionnaire of the Subjective Global Assessment (SGA), which aims to assess PEW on the basis of the patient’s history of nutrition quantity and quality combined with anthropometric measurements of fat and muscle tissue loss. The third was body composition analysis using BIA, which allows for the estimation of lean tissue and fat tissue mass as well as water content and its localization. The fourth area of interest was the laboratory markers of nutritional state: serum albumin, creatinine, and total cholesterol. Furthermore, we investigated the impact of DM on morbidity and mortality, with emphasis on CV complications.

The dietary analysis revealed that patients with DM received a caloric diet that exceeded their energy demands as reflected by total metabolic rate, while non-DM subjects obtained slightly less calories than required. Moreover, DM patients obtained significantly more proteins, lipids, and carbohydrates than those without DM. The DM group intake of these nutritional components also exceeded their calculated dietary requirements for body weight maintenance and was significantly richer in sodium.

The laboratory results showed comparable control of anemia, iron metabolism, inflammation, and bone-mineral disease. Higher CV morbidity and mortality of the DM group was accompanied by a higher median hs cTnT concentration. Despite the higher caloric intake of MHD recipients with DM, the serum indicators of nutritional state—albumin, creatinine, and total cholesterol—were significantly lower in this population.

For an in-depth analysis of the incompatibility between the higher energy intake and poorer nutritional state of DM patients as reflected by serum markers, we assessed body composition using BIA. The results showed no differences in LTM nor in LTI, for which decreased values have been proposed as one of the most valuable body composition indicators of PEW [24,25]. Nevertheless, it is also postulated that low muscle strength rather than low muscle mass is more strongly associated with PEW and the mortality of patients on chronic dialysis [26,27]. The higher median BMI and lower incidence of BMI < 23 kg/m^2^ in the DM group could be explained by the abundant intake of nutritional components, which led to a higher proportion of fat tissue reflected in the FTM and FTI, as well as excessive fluid measured as OH and relative OH. In the DM group, there was no negative correlation between cTnT and BMI or FTI, which was in fact detected in the Control group. This may suggest that these parameters of nutritional state are not as valuable as prognostic markers of PEW or CV risk for ESRD patients with DM as they are for the population without DM. The positive correlation for cTnT in the DM group was only observed for SGA and albumin, which may indicate that these markers have the highest predictive value in this specific subpopulation of patients receiving MHD. On the other hand, the significant negative correlation between cTnT and BMI, FTI, and total cholesterol in the Control group illustrates the previously described “reverse epidemiology” of classical CV risk factors in the ESRD population [28].

DM was connected with higher comorbidity, as reflected by the CCI, with a higher incidence of CHF, CAD, MI, CVI and the tendency for a higher incidence of HT. The presence of DM was a risk factor of higher CV and all-cause mortality. The interesting result obtained from our survival analysis is the distribution of overall mortality. The cumulative survival is comparable during the first 24 months of follow-up, whereas later, the survival curves split in favor of patients without DM. Patients from both groups remained on MHD for comparable periods of time, and during the first 2 years of follow-up, they demonstrated a similar risk of death. This may be explained by the previously demonstrated highest risk of death during the early period of MHD therapy [29].

Several previous studies have analyzed the nutritional state of ESRD patients with DM who are treated with MHD; however, the diagnostic methods were either sparse or different to the ones employed in our study. Some of these papers were not focused on DM as the crucial factor influencing nutritional state, whereas the others were performed on small cohorts, which significantly affected their statistical power.

One of the early studies by Pupim et al. monitored the indicators of malnutrition in ESRD patients initiating dialysis [18]. They analyzed the body mass composition, with albumin as a marker of nutritional state, and administered the SGA questionnaire. The studied measurements were determined once at baseline, i.e., the onset of dialysis, and then after 12 months. Their results showed a greater loss of LBM and lower serum albumin among patients with DM vs. non-DM. DM appeared to be the strong predictor of LBM loss. Both low albumin and greater loss of LBM may indicate DM as a potential risk factor of PEW in populations receiving MHD. The lower serum albumin in the DM population was concordant with our results. Nevertheless, due to the presentation of our baseline results of body composition measurements, we were not able to determine the LBM change over time.

A cross-sectional study by Boaz et al. analyzed the nutritional state of patients in Israel who were undergoing MHD according to the presence of DM. The malnutrition risk was assessed in terms of the presence of BMI < 23 kg/m^2^ or the serum albumin concentration < 38 g/L. They revealed a higher percentage of DM subjects than non-DM to be at risk of malnutrition. Moreover, albumin was lower in DM patients despite a higher BMI, revealing that malnutrition in DM patients on MHD may develop even in the presence of obesity [16]. These findings are in accordance with our results, promoting the hypothesis that the DM population on MHD is first and foremost at risk of body protein loss and serious body composition disorders such as lean tissue mass deficit and increased overhydration.

A study by Marcelli et al. aimed to assess the changes of body composition using bioimpedance spectroscopy during the early period of MHD. Body composition was repeatedly determined by BIA during the 2-year follow up. DM was significantly associated with a greater decrease in LTI and an increase in FTI [30]. Thus, this is another study that reflects the negative effect of DM on the body composition of MHD patients, demonstrating a more intensive loss of lean tissue than in patients without DM.

In contrast, a study by Visser et al. revealed different results. The study investigated 54 patients on MHD with high comorbidity and aimed to determine the presence and degree of muscle mass and muscle strength loss, as well as their nutritional status. The studied group was followed during 3–6 study visits. The results showed a decrease in lean tissue mass and handgrip strength, and an increase of fat tissue mass without any significant decrease in serum albumin concentration over time. DM was analyzed as a risk factor of muscle wasting. The DM patients’ mean LTM after 20 weeks was reduced by 23.3% vs. 14.1% in patients without DM. Nevertheless, the difference did not achieve statistical significance, most likely due to the small sample size. DM as a covariate in the multivariate model was also not associated with greater LTM loss over time [31]. We intend to verify the observations of Marcelli et al. and Visser et al. with serial BIA assessments during the follow-up of our studied group.

In another study by Mori et al., the muscle strength and mass of patients undergoing MHD was assessed. The diagnosis of sarcopenia was established in patients presenting with both low muscle strength and mass. DM was significantly more common in patients with sarcopenia than in those without, and it was an independent contributor to sarcopenia in patients undergoing HD [18]. Eventually, DM was classified as a risk factor of PEW, and low muscle mass and low muscle strength were emphasized as particularly valuable indicators for this risk prediction. Our results did not confirm the differences in LTM or LTI, and we did not assess muscle strength in our multifactorial analysis.

A small observational study performed on 21 DM patients receiving MHD assessed nutrition status with the employment of SGA. The SGA score categorized 47.6% of studied individuals as moderately malnourished and 9% as severely malnourished [32]. Unlike our analysis, this study did not investigate DM vs. non-DM populations, therefore it did not deliver any proof of DM as the risk factor of PEW that we suspected in our analysis.

Medeiros et al. studied 92 patients receiving MHD in Brazil to determine body composition, risk of sarcopenia, and serum sclerostin levels with respect to the presence of DM. The comparative analysis of subgroups with or without DM showed only the higher mean concentration of sclerostin among DM patients, without a significant difference in the incidence of sarcopenia [17]. This observation may to some degree support our findings, suggesting a higher risk of PEW development in ESRD patients on MHD with DM and may add another potential laboratory marker of malnutrition to the diagnostic panel. While ours and previous reports delivered various data supporting the hypothesis that there is an increased risk of PEW development in the population of MHD patients with DM rather than those without, the pathomechanism behind this phenomenon remains unclear. Previous studies have strongly suggested insulin resistance as a major factor promoting the catabolic pathway in ESRD [33,34,35]. Insulin resistance is a common complication in the course of CKD, a complex result of uremic toxicity, acidosis, chronic inflammation, and/or vitamin D deficiency, and it is not a disturbance specific for carbohydrate metabolism dysregulation [35,36,37,38]. Thus, the potentially higher risk of kidney disease-derived PEW in DM patients seems to require both the establishment of reliable diagnostic criteria and the determination of the pathomechanisms leading to its development. With our current knowledge, the therapeutic approach we recommend is the thorough holistic care provided by a multidisciplinary team, including but not limited to nephrologists, dieticians, endocrinologists, and general practitioners. Furthermore, there should be a strong emphasis on promoting physical exertion, optimal nutritional intake, and early qualification for kidney transplantation or simultaneous pancreas-kidney transplantation before the development of PEW.

Our study has several strengths. Firstly, it is one of the relatively few attempts to investigate the influence of DM on the nutritional state of ESRD patients on MHD. Secondly, it is a prospective study performed on a significant cohort of 515 patients, which lends significant statistical power to the presented data and thus a prominent position among other prospective observational studies in the field. Thirdly, it assesses the nutritional state of ESRD patients receiving MHD using several different diagnostic methods, including a thorough analysis of diet composition correlated with requirements, laboratory, anthropometric, and BIA assessment. The additional value is in our long-term survival analysis during the follow-up period.

The presented study has several limitations that require attention. Primarily, the presented data do not fully reflect the longitudinal character of the study. Our aim was to compare the results of the baseline assessment of our cohort’s nutritional state according to the presence or absence of DM, whereas some of the qualified patients underwent additional follow-up visits using the same protocol. We are planning to collect the additional data and present them in a future analysis of the parameters as they change over time. In addition, our study protocol did not include muscle strength determination, which is postulated as a valuable indicator of nutritional status in the studied population.

## 5. Conclusions

MHD recipients with DM experience overnutrition as evidenced by higher BMI, FTI, and energy intake, with a paradoxically higher predisposition to PEW as expressed by a higher SGA score and lower serum markers of nutrition, e.g., albumin, total cholesterol, and creatinine. Our results are in compliance with the previously described pathological phenomenon of higher PEW prevalence despite higher BMI, FTI, and caloric intake—known as obese sarcopenia—which is known to be more common in MHD patients with DM [39,40]. We did not confirm the previously presented lower LTI and LTM in patients with DM [19,30]. MHD recipients suffering from DM are also affected by the higher burden of CV comorbidity, which leads to a higher risk of death more than 2 years after MDH initiation. SGA and serum albumin seem to be valuable prognostic markers of CV risk for this group of patients.

## Figures and Tables

**Table 1:** Baseline characteristics of studied groups.

Parameter	Group DM	Control	*p* Value
*n*	198	317	
Age, years	68.7 (59.3–76.4)	67.0 (57.3–76.4)	0.48
Male Gender, *n* (%)	120 (60.6)	190 (59.9)	0.88
Duration of MHD, months	48.2 (31.4–69.4)	51.8 (33.0–85.9)	0.09
Smoking, *n* (%)	59 (29.8)	115 (36.3)	0.16
Alcohol intake, *n* (%)	53 (26.8)	82 (25.9)	0.95
Additive salt, *n* (%)	92 (46.5)	143 (45.1)	0.92
SGA score, points	12.0 (10.0–15.0)	11.0 (9.0–13.0)	*0.00054*
CCI, points	5 (4–6)	3 (2–4)	*<0.0001*
Residual diuresis, ml	725 (200–1000)	500 (100–1250)	0.98
Blood pressure, mmHg	140/80 (130/70–160/90)	140/80 (125/70–155/90)	0.45 for systolic/0.11 for diastolic
**Comorbidities:**			
Hypertension, *n* (%)	184 (92.9)	268 (84.5)	*0.053*
Chronic heart failure, *n* (%)	120 (60.6)	145 (45.7)	*0.017*
Coronary artery disease, *n* (%)	126 (63.6)	143 (45.1)	*0.00043*
Myocardial infarction, *n* (%)	65 (32.8)	58 (18.3)	*0.00024*
Cerebrovascular incident, *n* (%)	37 (18.7)	25 (7.9)	*0.00022*
Atrial fibrillation, *n* (%)	59 (29.8)	89 (28.1)	0.64
Death, *n* (%)	89 (44.9)	99 (31.2)	0.0022
Cardiovascular death, *n* (%)	59 (29.8)	60 (18.9)	0.0073

CCI = Charlson Comorbidity Index; DM = diabetes mellitus; MHD = maintenance hemodialysis; SGA = Subjective Global Assessment; values presented as median (Q1–Q3) unless stated otherwise; Numbers in italic mean statistical significance.

**Table 2:** Cox proportional hazards model.

Parameter	Hazard Ratio (HR)	95% HR Confidence Interval	*p*-Value
Age (in decades)	1.49	1.26–1.76	*<0.001*
Sex. F vs. M	0.74	0.51–1.09	0.13
Diabetes	1.65	1.11–2.43	*<0.05*
BMI	0.95	0.91–0.99	*<0.05*
SGA	1.09	1.03–1.14	*<0.01*
Albumin	0.65	0.44–0.97	*<0.05*
Hemoglobin	0.80	0.70–0.90	*0.001*
Anuria	2.45	1.64–3.67	*<0.001*

BMI = Body Mass Index; F = Female; M = Male; SGA = Subjective Global Assessment; Numbers in italic mean statistical significance.

**Table 3:** Laboratory parameters.

Serum Parameter	Group DM	Control	*p* Value
Hypoalbuminemia *, *n* (%)	53 (26.7)	75 (23.7)	*0.017*
Albumin, g/dL	3.93 (3.61–4.20)	4.10 (3.80–4.30)	*0.0035*
Creatinine, mg/dL	6.34 (5.08–7.33)	7.12 (5.70–8.51)	*0.042*
hsTnT, ng/ml	54 (37–101)	48 (29–81)	*0.018*
Total cholesterol, mg/dL	158 (133–196)	180 (148–206)	*0.0046*
WBC, ×10^3^/µl	6.90 (5.57–8.30)	6.70 (5.50–8.44)	0.69
Hemoglobin, g/dL	11.0 (10.1–11.8)	11.0 (10.1–12.0)	0.92
Sodium, mmol/L	139 (136–141)	139 (137–142)	0.18
Potassium, mmol/L	4.90 (4.40–5.40)	4.90 (4.47–5.50)	0.62
Calcium, mg/dL	8.53 (7.84–9.04)	8.60 (8.00–9.10)	0.64
Phosphate, mg/dL	5.20 (4.22–6.40)	5.11 (4.14–6.30)	0.70
iPTH, pg/mL	248 (153–414)	270 (167–531)	0.22
Iron, µg/dL	65 (46–85)	67 (50–84)	0.69
TIBC, µg/dL	225 (194–256)	212 (184–245)	0.082
TSAT, %	27.7 (23.3–39.7)	32.2 (24.0–41.0)	0.25
Ferritin, ng/mL	516 (216–1346)	609 (155–1090)	0.71
C-reactive protein, mg/L	5.7 (2.1–11.6)	5.3 (2.0–12.2)	0.99

* Hypoalbuminemia defined as <3.8 g/dL. hsTnT = high sensitivity cardiac troponin T; DM = diabetes mellitus; iPTH = intact parathormone; TIBC = total iron binding capacity; TSAT = transferrin saturation; WBC = white blood cells; Values presented as median (Q1–Q3) unless stated otherwise; Numbers in italic mean statistical significance.

**Table 4:** Body composition parameters.

Parameter	Group DM	Control	*p* Value
BMI, kg/m^2^	27.9 (24.4–31.8)	25.6 (22.9–28.8)	*<0.0001*
BMI < 23, *n* (%)	33 (16.7%)	80 (25.2%)	*0.0081*
LTM, kg	32.4 (26.3–38.5)	32.9 (26.9–41.0)	0.27
LTI, kg/m^2^	11.3 (10.0–13.3)	11.7 (10.0–13.8)	0.12
FTM, kg	31.6 (26.3–38.5)	25.85 (19.7–33.3)	*<0.0001*
FTI, kg/m^2^	15.0 (11.4–19.6)	12.8 (9.6–16.0)	*<0.0001*
OH, l	2.1 (1.2–4.1)	1.8 (0.7–2.7)	*0.00039*
Relative OH, %	3.0 (1.5–5.9)	2.5 (1.0–4.2)	*0.0059*

BMI = Body Mass Index; FTI = fat tissue index; FTM = fat tissue mass; LTI = lean tissue index; LTM = lean tissue mass; OH = overhydration; DM = diabetes mellitus; values presented as median (Q1–Q3) unless stated otherwise; Numbers in italic mean statistical significance.

**Table 5:** Nutritional parameters.

Parameter	Group DM	Control	*p* Value
Energy, kcal	2668 (2456–2760)	2279 (2122–2611)	*0.00084*
Total metabolic rate, kcal	2457 (2426–2599)	2391 (2218–2529)	0.24
Energy, %	107.8 (97.1–115.1)	96.7 (89.7–105.3)	*0.016*
Protein, g	92 (81–102)	79 (69–89)	*0.0063*
Required protein, g	86.7 (83.2–89.1)	82.0 (76.0–86.7)	0.25
Protein, %	112.2 (100.1–120.9)	96.4 (85.1–110.2)	*0.0063*
Lipids, g	82 (79–87)	75 (68–82)	*0.0033*
Required lipids, g	84.3 (80.9–86.6)	79.7 (73.9–84.3)	0.25
Cholesterol, mg	352 (321–398)	309 (221–365)	*0.033*
Lipids, %	100.3 (90.0–104.4)	93.4 (85.3–103.2)	0.11
Carbohydrates, g	376 (346–403)	321 (308–375)	*0.00095*
Required carbohydrates, g	345.9 (341.3–365.7)	336.4 (312.0–355.9)	0.25
Carbohydrates, %	109.9 (97.2–116.5)	99.5 (89.8–106.8)	*0.024*
Fiber, g	14.7 (14.0–17.0)	16.0 (14.0–18.0)	*0.29*
Na, mg	4581 (3879–5328)	3875 (3210–4501)	*0.034*
K, mg	3861 (3451–4351)	4142 (3502–4532)	*0.55*

DM = diabetes mellitus; values presented as median (Q1–Q3) unless stated otherwise; Numbers in italic mean statistical significance.

**Table 6:** Correlations for cTnT.

Parameter	Group DM r; *p* Value	Control r; *p* Value
Body Mass Index	0.11; 0.21	*−0.20*; *0.003*
Fat Tissue Index	0.14; 0.12	*−0.16*; *0.022*
Fat Tissue Mass	−0.09; 0.35	*−0.16*; *0.016*
Lean Tissue Index	0.08; 0.40	−0.11; 0.11
Lean Tissue Mass	0.14; 0.13	−0.10; 0.15
Albumin	*−0.23*; *0.016*	*−0.23*; *0.001*
Creatinine	−0.17; 0.44	0.11; 0.39
Total cholesterol	−0.16; 0.18	*−0.22*; *0.012*
Charlson Comorbidity Index	−0.26, 0.54	0.36; 0.10
Energy	0.19; 0.52	−0.23; 0.22
Energy%	−0.20; 0.48	0.01; 0.94
Subjective Global Assessment	*0.24*; *0.022*	*0.25*; *0.002*

Numbers in italic mean statistical significance.

## Data Availability

The data are not publicly available due to reasons of privacy.
